# Supplementary material for: MCT-Induced Ketosis and Fiber in Rheumatoid Arthritis (MIKARA)—Study Protocol and Primary Endpoint Results of the Double-Blind Randomized Controlled Intervention Study Indicating Effects on Disease Activity in RA Patients
Source: Nutrients. 2023 Aug 25;15(17):3719. doi: 10.3390/nu15173719 (PMC10490289; doi:10.3390/nu15173719)
Supplement: Supplementary file 1 [file nutrients-15-03719-s001.zip › Supplementary Materials Updated/Figure S1.pdf]

Figure S1: Record Template for BHB levels

MCT **I**nduzierte **K**etose versus Ball**A**ststoffe bei **R**heum**A**-Erkrankungen **(MIKARA)**

Messprotokoll
Blutglucose und Ketone

Studienarzt	Prof. Dr. med. Monika Reuss-Borst
Patienten-ID	

Liebe Patientin, lieber Patient,

Sie haben von uns einen mobilen, kompakten Blutzucker- und β-Keton-Messgerät „GlucoMen® areo 2K" erhalten. Die Bedienung wurde mit Ihnen im Vorbesprechungstermin besprochen. Das Gerät dient dazu, schnell und einfach Blutglukose und Ketone im Blut zu erfassen. Wir bitten Sie, damit zweimal täglich 30 Minuten nach der Einnahme des Nahrungs-Supplements den Ketonkörper-Spiegel und Blutglukose zu messen und protokolieren.

WOCHENDATUM: ____________________

	**KETONE (mmol/l)**		**BLUTZUCKER (mg/dl)**		
					30 Minuten nach Einnahme
Wochentag	Morgens	Nachmittags	Morgens	Nachmittags	
(Tag/Monat)	(mmol/l)	(mmol/l)	(mg/dl)	(mg/dl)	
(Tag/Monat)	(mmol/l)	(mmol/l)	(mg/dl)	(mg/dl)	
(Tag/Monat)	(mmol/l)	(mmol/l)	(mg/dl)	(mg/dl)	
(Tag/Monat)	(mmol/l)	(mmol/l)	(mg/dl)	(mg/dl)	
(Tag/Monat)	(mmol/l)	(mmol/l)	(mg/dl)	(mg/dl)	
(Tag/Monat)	(mmol/l)	(mmol/l)	(mg/dl)	(mg/dl)	
(Tag/Monat)	(mmol/l)	(mmol/l)	(mg/dl)	(mg/dl)	

Figure S1: Record Template for BHB levels
