# Supplementary material for: MCT-Induced Ketosis and Fiber in Rheumatoid Arthritis (MIKARA)—Study Protocol and Primary Endpoint Results of the Double-Blind Randomized Controlled Intervention Study Indicating Effects on Disease Activity in RA Patients
Source: Nutrients. 2023 Aug 25;15(17):3719. doi: 10.3390/nu15173719 (PMC10490289; doi:10.3390/nu15173719)
Supplement: Supplementary file 1 [file nutrients-15-03719-s001.zip › Supplementary Materials Updated/Table S1 Study Assessments.pdf]

Table S1: Study assessments at T0, T2 and T4 (MIKARA study)

VISIT	T0	T2	T4
**Time Point (study week)**	**0**	**8**	**16**
Informed Consent	x		
Eligibility	x		
Case history and Sociodemographics	x		
Medication	x	x	x
Physical examination	x	x	x
Vital signs	x	x	x
Anthropometric data	x	x	x
Urine sample	x	x	x
Blood sample	x	x	x
Stool sample	x	x	x
SDAI	x	x	x
DAS-28	x	x	x
Morning stiffness	X	X	x
HAQ	x	x	x
PHQ9	x	x	x
MFI	x	x	x
SF-36	x	x	x
FFkA	x	-	x
Sensory	x	x	x
Smoking, Coffee, Alcohol habits	x	x	x
3 day-food record	x	x	x
Adverse events (AE)	x	x	x
Adherence	x	x	x
BHB	x	x	x
