# Supplementary material for: MCT-Induced Ketosis and Fiber in Rheumatoid Arthritis (MIKARA)—Study Protocol and Primary Endpoint Results of the Double-Blind Randomized Controlled Intervention Study Indicating Effects on Disease Activity in RA Patients
Source: Nutrients. 2023 Aug 25;15(17):3719. doi: 10.3390/nu15173719 (PMC10490289; doi:10.3390/nu15173719)
Supplement: Supplementary file 1 [file nutrients-15-03719-s001.zip › Supplementary Materials Updated/Table S2 Primary and secondary endpoints.pdf]

Table S2: Primary and secondary endpoints

Primary Endpoint	• Score reduction of SDAI indices before (T0) and after intervention (T4); defined as T0 minus T4.
Secondary endpoints	• Change in disease activity in terms of DAS-28 (Disease Activity Score-28), ESR, CRP and morning stiffness (in minutes) before and after the intervention (T0, T2, T4).<br>• Change in functional status in terms of HAQ (Health Assessment Questionnaire) before and after the intervention (T0, T2, T4).<br>• Change in mood/depressiveness in terms of BDI (Beck Depression Inventory) and PHQ-9 (Patient Health Questionnaire-9) before and after the intervention (T0, T2, T4).<br>• Change in health-related quality of life with regard to SF-36 (Short Form-36) before and after the intervention (T0, T2, T4).<br>• Change in fatigue symptoms and physical activity with regard to MFI (Multidimensional Fatigue Inventory) and FFkA (Freiburg Physical Activity Questionnaire) before and after the intervention (T0, T2, T4).<br>• Changes in various blood values before and after intervention (T0, T2, T4) including:<br>1. Lipids, metabolic profile (CRP, TG, LDL, HDL, Chol, rheumatoid factor, creatinine, urea, potassium, sodium, blood count, diff.-BB, GPT, GOT, GGT).<br>2. Cellular measurements: distribution of different immune cell populations in peripheral blood mononuclear cells (PBMCs); activation levels of monocytes (M1 and M2) and T cells, Th1/T17 and, Treg cells, expression of adhesion molecules.<br>3. Inflammatory cytokines, factors and insulin including IL-1, HMGB-1, RAGE, IL-6, IL-18, Il-1beta, IL-17, TNF-alpha, Nesfatin-1, IL-35.<br>4. Redox status including lipoxygenases (LOX), cyclooxygenases (COX) and glutathione peroxidases (GPx) as well as their metabolites<br>• Change in the fecal microbiota during the intervention, including:<br>1. Fecal microbiome<br>2. Fecal fatty acid profile (butyrate, acetate, propionate, iso-butyrate, isovalerate)<br>3. Fecal biomarkers: Alpha 1-Antitrypsin, Zonulin, Calprotectin<br>• Change in clinical factors before and after intervention (T0, T2, T4) including BMI, weight, vital signs, Ketone bodies (beta-hydroxybutyrate), glucose, insulin.<br>• Analysis of sensory and nutritional factors that determine compliance and effectiveness of the intervention (T0, T2, T4).
